# Oncology training programmes for general practitioners: a scoping review

**DOI:** 10.3332/ecancer.2021.1241

**Published:** 2021-06-03

**Authors:** Bishal Gyawali, Matthew Jalink, Sophie Marie Anne Effing, Nancy Dalgarno, Klodiana Kolomitro, Niresh Thapa, Bishesh Sharma Poudyal, Scott Berry

**Affiliations:** 1 Division of Cancer Care and Epidemiology, Cancer Research Institute, Queen’s University, 10 Stuart Street, 2nd Level, Kingston, ON K7L 3N6, Canada; 2 Department of Public Health Sciences, Queen’s University, Kingston, ON K7L 3N6, Canada; 3 Department of Oncology, Queen’s University, 76 Stuart Street, Kingston, ON K7L 2V6, Canada; 4 Undergraduate Student, Queen’s University, Kingston, ON K7L 3N6, Canada; 5 Office of Professional Development and Educational Scholarship, Faculty of Health Sciences, Queen’s University, 18 Barrie Street, Kingston, ON K7L 3N6, Canada; 6 Karnali Academy of Health Sciences, Jumla, Military Sadak, Chandannath 21200, Nepal; 7 Clinical Hematology and Bone Marrow Transplant Unit, Department of Medicine, Civil Service Hospital, Kathmandu 44600, Nepal; † contributed equally

**Keywords:** oncology, general practice, medical oncology training, training, curriculum

## Abstract

**Introduction:**

Due to the increasing global burden of cancer and the shortage of trained medical oncologists, training General Practitioners (GPs) in Oncology (known as GPOs) has been proposed as a means to potentially ease some burden on medical oncologists with heavy workloads, especially in low-and-middle-income countries (LMICs), by task-sharing and task-shifting. We undertook a scoping review to identify and characterise the existing training programmes and curricula for GPOs globally.

**Design:**

We searched three major electronic databases: EMBASE, Medline/PubMed and Education Source for articles that described a medical oncology training programme for GPs. All study types were eligible in this review. We followed a two-stage standardised screening process using two independent reviewers to evaluate the eligibility of the articles.

**Results:**

Five peer-reviewed articles were included in our review and grey literature scans identified an additional seven GPO training programmes for a total of 12 programmes and their curricula. All of the included studies were from high-income countries. The duration of programmes varied from comprehensive programmes structured over 2 years (*n* = 2) to shorter duration medical oncology training activities (*n* = 2), a short, 1.5-day workshop and a 10-hour course. In the grey literature, GPO training programme durations ranged from 2 weeks to 13 months. A mixture of delivery methods was employed including didactic lectures and clinical rotations.

**Conclusion:**

This scoping review identified a small number of heterogeneous studies and grey literature sources that described and/or evaluated medical oncology training programmes for GPs. The information synthesised here can be used to foster the collaboration needed for the continued development of GPO programmes that could help address the problem of lack of workforce to meet the rising burden of cancer, especially in LMICs.

## Introduction

The rising incidence of cancer is a major global issue, challenging nations’ socioeconomic development and healthcare systems [1]. Global cancer cases will increase from 17.0 million to 26.0 million between 2018 and 2040 [1]. The corresponding number of patients requiring first-course chemotherapy annually during this time frame will rise from 9.8 million to 15.0 million, a relative increase of 53% [1]. The burden of cancer is disproportionately high in low-and-middle-income countries (LMICs), where 63% of cases occurred in 2018, and will increase to 67% in 2040 [1]. This disproportionate cancer burden is further compounded by the lack of cancer physicians in LMICs [2]. Unfortunately, LMICs lack the workforce and resources needed to tackle this growing burden. Global surveys of medical oncologists have revealed substantial disparities in oncology workload between high-income countries (HICs) and LMICs with oncologists in LMICs working longer hours, caring for more patients and having lesser job satisfaction [3, 4]. By 2040, it is predicted the world will need an additional 100,000 cancer physicians, most of who would be needed in LMICs [1].

Becoming a cancer physician requires sub-speciality training that typically takes 5–6 years of post-graduate training [5]. Training of such specialists to take care of the growing cancer burden in LMICs is resource intensive and time consuming. Highly focused training programmes are not often available in LMICs. This leads to significant challenges in training new, qualified cancer physicians to provide care for the rising number of patients [1]. Until adequate specialists are trained and available, an alternative is needed. In many LMICs, doctors who are trained in internal medicine or general surgery or paediatrics can also take up additional months of specialised training to get some experience in delivering chemotherapy to patients with cancer. However, these physicians do not necessarily work in collaboration with oncologists, nor do they usually work in remote locations to compensate for the lack of an oncology provider.

A potential measure to help address this problem would be to train General Practitioners (GPs), also known as primary care physicians, in the foundations of oncology care. This concept of task-shifting was pivotal in the scaling up of successful HIV care in the 2000s and has been proposed as a model to meet the growing need for oncology care in LMICs [6]. Due to the high prevalence of cancer globally, many GPs are involved with managing cancer care and will continue to play a significant role in coordinating care [7]. GPs play an important role in the management of patients with cancer since patients first present to their GPs for screening or with initial symptoms of cancer and continue to be followed up by them after primary treatment of cancer. Previous experiences in LMICs have shown that GPs can safely deliver chemotherapy provided with adequate remote support from specialist centres [8].

General Practitioners in Oncology (GPOs) are specially trained family doctors with a clinical focus on oncology [9]. These physicians dedicate their clinical practice specifically to oncology on a significant part-time or full-time basis [9]. A wide array of roles has been described for GPOs, and these roles vary substantially depending on the practice is in an urban or rural setting [7]. Common roles include the clinical supervision of systemic therapy, management of treatment-related physical and psychosocial effects, provision of cancer survivorship follow-up care and palliative medicine [7]. GPOs can play a significant role in rural communities, where cancer care and treatment is scarce. The existing peer-reviewed evidence describing GPO training programmes is widely dispersed and, to the author’s knowledge, has not yet been synthesised.

However, there are no formal programmes and curricula designed to train GPs in chemotherapy delivery in these settings. We undertook a scoping review to identify and characterise the existing evidence for medical oncology training programmes and curricula for GPOs across the world in low, middle and high-income settings.

## Methods

### Scoping review explanation and rationale

While systematic reviews summarise all available empirical evidence on a narrow, focused topic, scoping reviews facilitate the exploration and examination of a broad topic of interest with the purpose of identifying gaps in the evidence, clarifying concepts and synthesising what is known about a topic [10]. Although conducted for different purposes compared to systematic reviews, scoping reviews still require rigorous and transparent methods in their conduct to ensure the validity of the results [11]. We chose this methodology due to the lack of empirical literature on this subject and to capture all available information regarding medical oncology training programmes for GPs. We focused on medical oncology training programmes since surgical and radiation oncology programmes require specialised skillsets and infrastructure and are therefore considered beyond the scope of a GPO. The framework developed by Arksey and O’Malley (2005) was used for this scoping review [12]. Our research questions included: 1. What are the available medical oncology training programmes and/or curricula for GPs in oncology? 2. What are the characteristics of these medical oncology training programmes?

### Data sources and search strategy

A systematic search strategy was developed and executed in three major electronic databases: EMBASE, Medline/PubMed and Education Source (Supplementary Appendix). The reference lists of relevant literature review papers and included articles were scanned for additional eligible articles. It is also common for such training programmes to not be published in a peer-reviewed literature but simply posted on the institution’s webpage or internal documents. Such information produced outside of traditional publication and distribution channels are called ‘Grey literature’. We scanned Grey literature sources such as unpublished curriculum at different academic institutions or at professional organisations using their respective websites.

### Eligibility criteria

The population of interest were GPs who underwent medical oncology training of any kind. Medical oncology training was defined as any of the following: an accredited postgraduate programme at a medical school, continuing medical education initiative, certificate programme and/or a clinical placement programme. Articles were evaluated if they included our population of interest and described a medical oncology training programme for GPs in all countries and income settings. Only English- and French-language articles were considered. All study types were included in this review with the exception of conference abstracts and any articles that did not adequately describe the content of the medical oncology training component. Review articles were also excluded, but the reference lists of relevant reviews were hand-searched for additional eligible articles.

### Screening of studies and data extraction

We followed a two-stage standardised screening process to evaluate the eligibility of the articles identified in the search using Covidence systematic review software [13]. Two independent reviewers (MJ, SE) screened the title and abstracts of the articles using the developed eligibility criteria. During the second stage, the same reviewers independently screened the full text articles that passed the stage 1 review. Disagreements were resolved through discussion and consensus between the two reviewers, and consultation with the third reviewer (BG).

Data were extracted using a pre-developed data extraction tool which included the terms: author, year, country setting, population(s), programme name, training programme description, duration, method of delivery, clinical rotation (Y/N), number of programme participants, formal curriculum (Y/N), recommended textbook or reference, outcomes measured and general comments. The data extraction tool was developed specifically for this review.

### Synthesis

Extracted data is displayed in Table 1. Trends and patterns found in the final eligible study list were evaluated. Due to the small number of eligible studies, thematic analyses were restricted. Quantitative synthesis was not possible due to the nature of the review and heterogeneity in the included studies. A narrative synthesis of the available training programmes was conducted.

## Results

**Figure 1.**
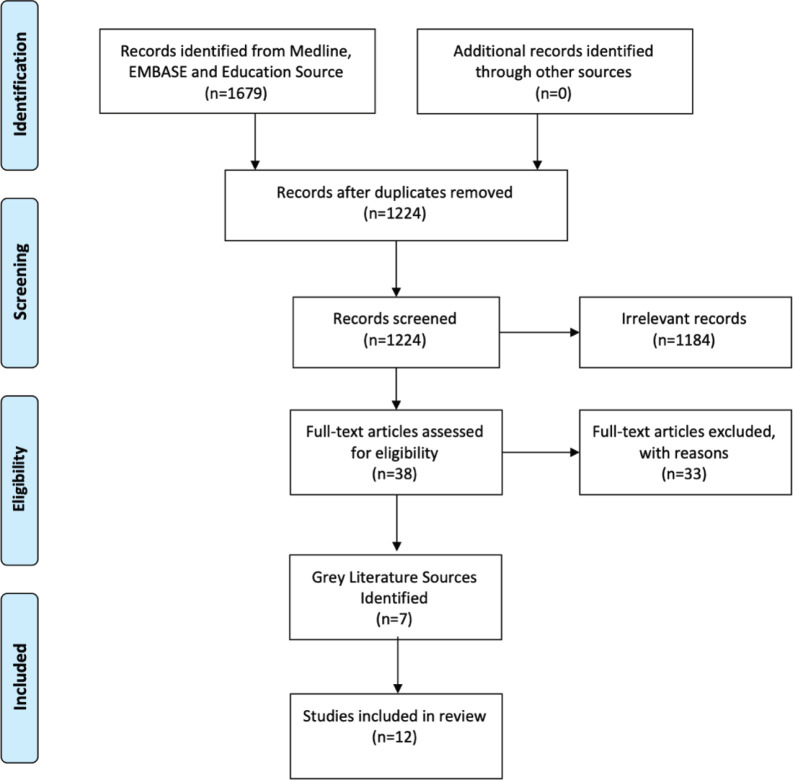
PRISMA flow diagram.

The initial search yielded 1,679 articles, of which 38 articles were reviewed in full and five peer-reviewed articles were included in our review as depicted through the Preferred Reporting Items for Systematic Reviews and Meta-Analyses (PRISMA) flowchart in Figure 1. Grey Literature scans identified an additional seven GPO training programmes for a total of 12 programmes and their curricula included in this synthesis (Table 2).

### Location of programmes

All of the included studies were from HICs (Australia, The Netherlands, Sweden and the United States). The predominant population evaluated GPs and GP trainees. Most programmes were based in urban centres, though one study (Dalton *et al* [16]) specifically looked at rural prescribers of chemotherapy. All of the curricula identified through grey literature search were from institutions in Canada, except for one curriculum from the University of Porto in Portugal.

### Programme content and duration

Two articles described comprehensive programmes that were structured over 2 years as continuing medical education courses covering a range of topics including prevention, early diagnosis, treatment of side effects, rehabilitation, follow-up and palliative care [14, 15]. Two articles described shorter duration medical oncology training activities [16, 17]. The study by Dalton *et al* [16] described a short, 1.5-day workshop that covered the role of adjuvant chemotherapy, managing the side effects of chemotherapy, safe administration of cytotoxic drugs, endocrine therapy, communication skills and providing psychological support to equip rural GPs. The second study from Australia by Evans *et al* [17] described a 10-hour clinical placement at a cancer centre that focused on the role of shared care in post-treatment cancer and mechanisms to strengthen links between generalist and specialist care providers. Another described the teaching methods used in American GPO programmes for cancer pain management but did not report on the duration of the programme [18].

In the grey literature, GPO training programme durations ranged from 2 weeks to 13 months. Only three studies reported programme cohort sizes, which ranged from 28 to 33 participants [15–17]. Two of the seven studies identified in the review contained formal curricula, and none referenced a textbook or other course resources. Common clinical rotation blocks included: inpatient medical oncology, common solid tumour sites (lung, breast, gastrointestinal, melanoma/head & neck, genitourinary), haematology, gynaecologic oncology, radiation oncology and palliative care.

### Method of delivery

In the five studies identified in the literature search, a mixture of delivery methods was employed. An American study discussed didactic lectures, interactive question/answer periods, small group case-based discussions and role-playing exercises [18]. The 1.5-day workshop described in Dalton *et al* [16] only used didactic presentations. The 10-hour in-hospital placement programme described in Evans *et al* [17] did not include any classroom instruction. The remaining two studies (Carlsson *et al* [15] and Schadé [14]) evaluated programmes that included a combination of both didactic lectures and clinical rotations. All programmes identified in the grey literature search used different combinations of clinical rotations and didactic lectures.

Of the programmes identified in the grey literature, all integrated clinical rotations in their curriculum with the exception of the three-day postgraduate short course by the University of Porto, Portugal, which used patient simulations. Didactic lectures supplemented clinical rotations in all programmes.

Three of the five peer reviewed studies [15–17] evaluated outcomes, which focused on knowledge gained using pre- and post-education tests to determine the efficacy of the programmes. Evans *et al* [17] found that the programme’s learning outcomes, personal learning needs and relevance to practice were entirely or partially met, and all respondents would recommend the programme due to the applicability of the knowledge and skills learned. Dalton *et al* [16] found significant positive changes in participants’ perceived knowledge after completing GPO training. These areas of improvement included: knowledge and occupational health and safety surrounding the prescription of cytotoxic drugs (2.54 (low-moderate) to 3.76 (moderate to high), prevention of side effects (3.07–3.88) and psychological consequences 2.94–3.73)) [16]. Carlsson [15] found significant improvements in the domains of knowledge (mean difference: 2.6, 95% confidence interval (CI): 1.3–3.8) and attitudes (2.9, 95% CI: 0.8–5.0), but not for patient management (0.3, 95% CI: 0.6–1.2)). No grey literature sources evaluated outcomes.

### A model of task-shifting from a LMIC

During our literature search, although not fulfilling our criteria for inclusion, we found the description of a programme from LMIC that provides some important insights into the feasibility of a GPO programme in LMIC. The Butaro Cancer Center of Excellence in rural Rwanda, in collaboration with Partners in Health/Brigham and Women’s Hospital, has provided support to non-oncology providers (physicians and nurses) in the care of patients with cancer including diagnosis, treatment and follow-up since 2012 [6]. The use of agreed upon treatment pathways and the provision of continuous, reliable, structured virtual consults with trained oncology professionals from the U.S alongside frequent on-site visits have been highlighted. Although this does not represent a formal training curriculum for a fixed duration, the focus of our review, this does provide important insights into a long-term practical and sustainable model for global North-South collaboration in this domain.

## Discussion

The purpose of this scoping review was to identify and describe the available empirical and grey literature related to medical oncology training programmes for primary care providers. Consolidating this heterogenous literature allows to understand different types of training programmes in the world for GPOs and to learn from those experiences. A key finding is the scarcity of peer-reviewed literature from LMICs that highlights the need to adapt programmes from HIC or develop programmes in LMICs to address the need for an increased workforce for the growing cancer burden in these countries.

In the five identified studies, there was little similarity regarding the structure, formation and content of the GPO training programmes. In the seven grey literature programmes, more similarities were observed as all programmes delivered their content using both didactic lectures and clinical rotations, however, all these programmes were located in the same country with the exception of one. The programmes differ in terms of content and mode of delivery and duration as well as outcomes of the programme. These data may help other institutions planning to develop similar training programmes in the future.

Didactic modes of teaching remain the most common mode of training, present in all but one programme identified in our review, followed by an integration of didactic teaching with clinical rotations. Experiences during the pandemic have demanded the delivery of didactic teaching and other educational material using online learning management platforms. These experiences could make the training programmes more appealing to institutions in LMICs and facilitate expertise exchange with programmes in HICs and co-development of educational materials. However, the clinical rotations will require on-site expertise and local infrastructure development to allow for placement and mentorship of trainees.

The content of the curriculum varies widely between the programmes, likely due to different jurisdictions having different needs and expectations of GPOs in their system of cancer care delivery. Every institution planning to develop a GPO training programme should carefully consider the potential roles and expectations from GPOs post training and plan the contents of the curriculum accordingly. Screening for cancer, active surveillance and follow-up after treatment for certain cancers, supportive care and pain management are vital components of any GPO training programme. However, including information on chemotherapy regimens for various tumour types would depend on the workload expectation of the GPOs. There is a textbook written by Dr Kelechi Eguzo, a Nigerian GPO, in collaboration with Canadian oncologists [19] that is tailored for training primary care physicians in the basics of oncology. A modified version that was adapted for local needs and contexts would be a valuable curricular resource for GPO training in LMICs.

The three studies that explored significant differences in knowledge using pre- and post-scores revealed short-term gains in knowledge, despite different formats, structures and delivery methods. This needs to be cautiously interpreted due to the limited amount of evidence provided. None of these studies, however, have assessed long-term outcomes related to knowledge retention, application of skills or improved patient outcomes, which remains a major limitation.

We are developing a GPO curriculum tailored to the context of a LMIC, Nepal, with engagement and input from all relevant stakeholders. This is a key step in the planning process to run such a training programme to meet the needs of cancer care in Nepal. Such a curriculum, when developed and published, may fill in the gap for the existing lack of training programme curricula in GPO from LMICs, and could be a model for other LMICs.

One major concern that could preclude adoption of GPO programmes globally is the potential fear of impact on quality of care. Unfortunately, none of the included studies measured patient satisfaction and outcomes as a metric of the training programme’s success. Furthermore, in some settings, GPO training programmes could be viewed upon by the oncologists’ community as replacing the need for specialised oncologists with less-rigorously trained doctors. However, since the objective of the GPO programme would not be to replace oncologists but to assist them by task-shifting and task-sharing, and an oncologist would continue to be responsible for treatment decisions, such concerns can be addressed. For a GPO training programme to be implemented successfully, we recommend that any future training programmes include an evaluation of intended long-term outcomes such as an increased number of GPOs, increased collaboration among GPs and oncology specialists, improved patient outcomes, increased job satisfaction among GPOs and increased patient satisfaction with their care. It is important to incorporate GPO training programmes in any country not with a view of a temporary band-aid solution to address the increased needs of cancer doctors, but as a sustainable way to continue to provide good quality, equitable and accessible care for patients with cancer. Since even HICs are incorporating the model of GPOs in cancer care, this model should be considered as a long-term action plan to be implemented in addition to – not instead of – continued and long-term investment in training oncologists.

### Limitations

There are some limitations to this scoping review. The absence of literature from LMICs is a key limitation. While in HICs, GPOs are commonly employed in large academic centres to reduce the workload of busy oncologists, GPOs are expected to fill in for the lack of oncologists in many LMICs. Thus, the curricula devised in the context of HICs, that assumes an oncologist and multi-disciplinary team are available for support, may not be fully applicable in LMICs. Although it may be possible that some participants of the GPO programmes described here were from LMICs, that data was not readily available. Tailoring the content based on needs assessments and adaptation to local contexts will be critical when developing such a curriculum for LMICs. A blind copy-paste of the curriculum from a different context will defeat the purpose.

Despite our deliberately broad search criteria, given that we limited our search to English and French language studies and excluded conference abstracts, some relevant studies may not have been included. Searching other databases or using other languages may have identified additional relevant articles such as the curricula that may have been written in the local language only.

## Conclusion

With the rising global burden of cancer and corresponding need for physicians who can administer chemotherapy, GPOs may prove to be a critical component to the ongoing search for a solution. This scoping review identified a small number of heterogeneous studies that described and/or evaluated medical oncology training programmes for GPs. Within the identified studies, delivery of the programme content through a combination of didactic lectures (both in-person and online) and clinical rotations was consistent, and further supported by grey literature sources. Important and relevant information regarding programme structure, duration, content and method of delivery have been synthesised and will help inform institutions planning to design such a programme in the future. The information synthesised here can be used to foster the collaboration needed for the continued development of GPO programmes that could help address the problem of lack of workforce to meet the rising burden of cancer in LMICs. The limited, fragmented and diverse landscape of GPO training programmes and literature describing and evaluating these programmes suggests that more peer-reviewed research and evaluation is needed in this area.

## Conflicts of interest

None.

## Funding

This work was supported by the Global Oncology Young Investigator Award 2020 to Bishal Gyawali, MD, PhD.

## Authors’ contributions

All authors have made substantive contributions to the study according to the Credit Taxonomy.

## Figures and Tables

**Table 1.** Studies evaluating GP in oncology programmes (peer-reviewed studies).

First author (year)	Setting	Population(s)	Programme name (if applicable)	Duration	Number of programme participants per year	Didactic lectures (Y/N)	Clinical rotation (Y/N)	Simulation /case-based (Y/N)	Formal	Training programme content	Outcome measures
NA [18]	USA	Medical students, postgraduate physicians training in primary carespecialities	NR	NR	NR	Y	NR	Y	N	Pain management and treatment, the importance of interdisciplinary teams and the individualisation of drug and non-drug treatments. The primary goals are to reinforce attitudes and skills learned during medical school and acquire more advanced knowledge.	NR
Carlsson *et al* [15]	Sweden	GP trainees	NR	2-years PT	33	Y	Y	Y	N	Emphasised those areas within oncology where primary care has a major role: prevention, early diagnosis, treatment of side effects of oncologic therapy, rehabilitation, follow-up and palliative care. Breaking bad news and the psychosocial aspects of cancer care, and the supporting role of the GP also covered.	Evaluated the knowledge, skills, and attitudes
Dalton *et al* [16]	Australia	Rural prescribers of chemo (GPs, Med Oncs)	NR	1.5 days	30	Y	N	N	Y	The role of adjuvant chemotherapy, managing the side effects of chemotherapy, endocrine therapy, safe administration and handling of cytotoxic drugs and their related wastes, legal issues, communication skills, psychosocial support and identification of local issues.	Knowledge pre- and post-scores
Evans *et al* [17]	Australia	GPs and GPNs	NR	10 hours	16 GPs and 12 GPNs	N	Y	N	N	The role of shared care in post-treatment cancer care and mechanisms to strengthen links between generalist and specialist cancer care providers. Relate the decisions made at diagnosis and during active treatment to the impact on cancer survivorship and posttreatment care in primary care.	Post-placement evaluations
Schadé [14]	The Netherlands	GPs	Oncology for GPs	2 years	NR	Y	Y	Y	Y	Primary topics included: 1. Prevention, 2. Detection, 3. Diagnostics, 4. Therapy, 5. Care and control.	NR

NA, Not applicable; NR, Not reported, GP, General practitioners; GPN,General practice nurses, Med Oncs, Medical oncologists; Y, Yes; N, No, PT, Part time

**Table 2.** GPOs programmes identified in grey literature.

Institution	Setting	Population(s)	Programme name (if applicable)	Duration	Number of programme participants per year	Method of delivery			Formal curriculum (Y/N)
						Didactic lectures (Y/N)	Clinical rotation (Y/N)	Simulation/case-bases	
University of Western Ontario	Canada	GPs	Family Practice-Oncology	13 months	NR	Y	Y	N	Y
CancerCare Manitoba	Canada	GPs	NR	2–4 weeks	NR	Y	Y	N	N
University of Manitoba	Canada	GPs	NR	6–12 months	NR	Y	Y	N	Y
University of Ottawa	Canada	GPs	Oncology for Family Residents	6–12 months	NR	Y	Y	N	Y
BC Cancer/UBC	Canada	GPs	General Practitioner in Oncology Education Program	8 weeks	NR	Y	Y	N	Y
University of Toronto	Canada	GPs	Enhanced Skills Program: Medical Oncology	1 year	NR	Y	Y	N	Y
University of Porto	Portugal	GPs, oncologists	2nd Postgraduate course in Oncology for General Practitioners and Young Oncologists	3 days	NR	Y	N	Y	N

BC, British Colombia; GP, General Practitioner; NR, Not reported; Y, Yes; N, No; UBC, University of British Columbia

## Appendix. Search criteria

### EMBASE

1. exp general practitioner/
2. exp primary medical care/
3. general practitioner.mp.
4. primary medical care.mp.
5. primary health care/
6. primary healthcare.mp.
7. primary care.mp.
8. primary health care.mp.
9. general practice/
10. general practi*.mp.
11. general practice physician.mp.
12. family physician*.mp.
13. general practice/
14. general practice.mp.
15. family practi*.mp.
16. family doctor*.mp.
17. 1 or 2 or 3 or 4 or 5 or 6 or 7 or 8 or 9 or 10 or 11 or 12 or 13 or 14 or 15 or 16
18. chemotherapy/
19. chemotherapy.mp.
20. chemo*.mp.
21. adjuvant chemotherapy/
22. adjuvant chemotherapy.mp.
23. cancer chemotherapy/
24. cancer chemotherapy.mp.
25. neoadjuvant chemotherapy/
26. neoadjuvant chemotherapy.mp.
27. oncology/
28. medical oncology.mp.
29. oncology.mp.
30. clinical oncology.mp.
31. systemic therapy/
32. 18 or 19 or 20 or 21 or 22 or 23 or 24 or 25 or 26 or 27 or 28 or 29 or 30 or 31
33. education/
34. educat*.mp.
35. curriculum/
36. curricul*.mp.
37. teaching/
38. teach*.mp.
39. training/
40. train*.mp.
41. 33 or 34 or 35 or 36 or 37 or 38 or 39 or 40
42. 17 and 32 and 41

### Medline

1. Physicians, Primary Care/
2. primary care.mp.
3. primary medical care.mp.
4. Primary Health Care/
5. primary health care.mp.
6. primary healthcare.mp.
7. General Practitioners/
8. general practi*.mp.
9. Physicians, Family/
10. family physician*.mp.
11. Family Practice/
12. family practi*.mp.
13. family doctor*.mp.
14. 1 or 2 or 3 or 4 or 5 or 6 or 7 or 8 or 9 or 10 or 11 or 12 or 13
15. chemotherapy.mp.
16. chemo*.mp.
17. Medical Oncology/
18. medical oncology.mp.
19. clinical oncology.mp.
20. systemic therapy.mp.
21. 15 or 16 or 17 or 18 or 19 or 20
22. exp Education/
23. educat*.mp.
24. curricul*.mp.
25. teach*.mp.
26. train*.mp.
27. 22 or 23 or 24 or 25 or 26
28. Telemedicine/
29. telemedicine.mp.
30. e-health.mp.
31. Remote Consultation/
32. remote consultation.mp.
33. 28 or 29 or 30 or 31 or 32
34. 14 and 21 and 27

### Ed Source

(family doctor or general practitioner or general practice) AND (cancer or oncolog* or chemo*)
